# Cardiac GRK2 Protein Levels Show Sexual Dimorphism during Aging and Are Regulated by Ovarian Hormones

**DOI:** 10.3390/cells10030673

**Published:** 2021-03-17

**Authors:** Alba C. Arcones, Melanie Raquel Martínez-Cignoni, Rocío Vila-Bedmar, Claudia Yáñez, Isabel Lladó, Ana M. Proenza, Federico Mayor, Cristina Murga

**Affiliations:** 1 Departamento de Biología Molecular and Centro de Biología Molecular Severo Ochoa (CBMSO) UAM-CSIC, Universidad Autónoma Madrid, 28049 Madrid, Spain; aconcepcion@cbm.csic.es (A.C.A.); rocio.vila@urjc.es (R.V.-B.); claudia.yannezb@estudiante.uam.es (C.Y.); fmayor@cbm.csic.es (F.M.J.); 2 Instituto de Investigación Sanitaria Hospital Universitario La Princesa and CIBER Cardiovascular (CIBERCV), ISCIII, 28028 Madrid, Spain; 3 Departament de Biologia Fonamental i Ciències de la Salut, Institut Universitari d’Investigació en Ciències de la Salut (IUNICS), Universitat de les Illes Balears, Institut d’Investigació Sanitària Illes Balears (IdISBa), 07122 Palma, Spain; mr.martinez@uib.es (M.R.M.-C.); isabel.llado@uib.es (I.L.); ana.proenza@uib.es (A.M.P.); 4 Departamento de Ciencias Básicas de la Salud, Área de Bioquímica y Biología Molecular, URJC, 28922 Madrid, Spain; 5 CIBER de Fisiopatología de la Obesidad y Nutrición (CIBEROBN), 28029 Madrid, Spain

**Keywords:** sexual dimorphism, cardiovascular disease, G protein-coupled receptor kinase 2, estrogens, mitochondria

## Abstract

Cardiovascular disease (CVD) risk shows a clear sexual dimorphism with age, with a lower incidence in young women compared to age-matched men. However, this protection is lost after menopause. We demonstrate that sex-biased sensitivity to the development of CVD with age runs in parallel with changes in G protein-coupled receptor kinase 2 (GRK2) protein levels in the murine heart and that mitochondrial fusion markers, related to mitochondrial functionality and cardiac health, inversely correlate with GRK2. Young female mice display lower amounts of cardiac GRK2 protein compared to age-matched males, whereas GRK2 is upregulated with age specifically in female hearts. Such an increase in GRK2 seems to be specific to the cardiac muscle since a different pattern is found in the skeletal muscles of aging females. Changes in the cardiac GRK2 protein do not seem to rely on transcriptional modulation since *adrbk1* mRNA does not change with age and no differences are found between sexes. Global changes in proteasomal or autophagic machinery (known regulators of GRK2 dosage) do not seem to correlate with the observed GRK2 dynamics. Interestingly, cardiac GRK2 upregulation in aging females is recapitulated by ovariectomy and can be partially reversed by estrogen supplementation, while this does not occur in the skeletal muscle. Our data indicate an unforeseen role for ovarian hormones in the regulation of GRK2 protein levels in the cardiac muscle which correlates with the sex-dependent dynamics of CVD risk, and might have interesting therapeutic applications, particularly for post-menopausal women.

## 1. Introduction 

Cardiovascular disease (CVD) constitutes a global burden and stands as the leading cause of morbidity and mortality worldwide [1,2]. Specifically, among women, it is the premier cause of death in the United States [3]. Human studies and experiments performed in animal models have revealed the occurrence of sex-biased sensitivity to the development of CVD. Incidences of cardiovascular pathologies are lower in pre-menopausal women compared to age-matched men. However, this apparent protection is lost with age, particularly after menopause [4,5,6,7]. The systemic decline in estrogen levels after menopause, such as 17 β-estradiol (E2) which is the major estrogen form, has been related to an increased risk of CVD development with age in women, as supported by hormone replacement data from animal models [8,9,10,11] and early post-menopausal patients [12,13,14,15]. Among the mechanisms implicated in age-dependent sensitivity to CVD, the mitochondrial dynamics stand out as a key modulator of cardiac output [16,17,18]. In particular, increased mitochondrial fusion correlates with better mitochondrial functionality and cardiac health in young females [10,19] and, conversely, a lower fusion ability is associated with decreased cardiac functionality [20,21].

G protein-coupled receptor kinase 2 (GRK2) plays an essential role in regulating cardiovascular physiopathology by controlling cardiac contractility and inotropy, while also modulating the mitochondrial dynamics and metabolism of the heart, given its unique ability to impinge on both the G protein-coupled receptor (GPCR) signaling cascades and the modulating non-canonical pathways [22,23]. Importantly, dysregulation of the GRK2 levels is linked with diseased states in different cardio-metabolic conditions [24,25]. For instance, increased levels of GRK2 mRNA and protein activity have been reported in the heart in experimental models of CVD [23,26]. The genetic deletion or pharmacologic inhibition of GRK2 confers protection against heart failure, cardio-metabolic dysregulation, and vascular dysfunction [22,27,28,29,30,31]. The mRNA levels of cardiac GRK2 appear to run in parallel with protein quantity in the cardiac tissue and peripheral blood cells of both CVD patients and murine models of disease [26,32]. GRK2 protein levels are tightly controlled by proteasomal [26,33] or autophagic degradation [34] in different tissues and in experimental settings. Recently, GRK2 has been shown in murine models to be altered in a sexually dimorphic fashion in the liver, white adipose tissue, and skeletal muscle [35]. Nonetheless, the detailed mechanisms implicated in the modulation of GRK2 protein levels in the heart remain to be characterized, especially in females. 

We herein describe unforeseen sex- and age-dependent patterns of GRK2 modulation in the murine heart which parallel the differential sensitivity described in the development of CVD. Age-dependent GRK2 protein upregulation in females can be recapitulated by the loss of ovarian hormones and are partly reverted by estrogen supplementation, mediators that may lay at the basis of the sexual dimorphism observed in cardiac GRK2 dynamics.

## 2. Materials and Methods 

### 2.1. Animal Protocols 

Experiments were performed using young (~4 months old) and aged (~15 months old) male and female C57 BL/6 J mice and Wistar rats. Animals were bred at a room temperature of 22 ± 2 °C on a 12:12 light–dark cycle (lights on at 08:00 a.m.) with a relative humidity of 50 ± 10% and under pathogen-free conditions in the animal facility of the Centro de Biologia Molecular Severo Ochoa with free access to food and water. Mice and rats were euthanized under fed conditions by cervical dislocation and cardiac and skeletal muscle were dissected. All animal experimentation procedures conformed to the European Guidelines for the Care and Use of Laboratory Animals (Directive 86/609) and were approved by the Ethical Committees for Animal Experimentation of the Universidad Autónoma de Madrid (PROEX 48/15).

### 2.2. Ovariectomy Model

Ovariectomy experiments were performed in female rats at Universitat de les Illes Balears as previously described [10]. Briefly, control female Wistar rats and ovariectomized rats were purchased from Charles River (Barcelona, Spain), where ovariectomies (OVX group) and sham surgeries (control group) were performed at 5 weeks of age. At ten weeks of age, OVX rats were divided into two experimental groups regarding their treatment with estrogens: the OVX group was treated with 17 β-estradiol (OVX + E2), administered by subcutaneous injection of 10 mg/kg of E2 every 48 h for 4 weeks previous to sacrifice (at 14 weeks of age); and the sham group (OVX) was treated with corn oil (vehicle).

### 2.3. Western Blotting

Mice or rats were euthanized by cervical dislocation or decapitation, respectively, and the hearts and skeletal muscles (soleus and gastrocnemius) were surgically removed, washed, dried, and frozen immediately in liquid nitrogen. Approximately 3 mm^3^ of the heart and ¼ of the dissected skeletal muscle (soleus and gastrocnemius) were homogenized in hypotonic buffer with a Triton X-100 (150 µL and 400 µL of lysis buffer were used for the heart and muscle, respectively) using metal beads in a Tissue Lyser with two 2 min pulses of 1/30 s speed (Qiagen, Hilden, Germany) as previously described [30]. A total protein quantity of 50 μg was resolved per lane by an SDS-PAGE and transferred to a nitrocellulose membrane.

Blots were probed with specific antibodies against GRK2 (sc-562, Santa Cruz, Dallas, TX, USA), OPA-1 (optic atrophy 1, #612606, BD Transduction Laboratories, Franklin Lakes, NJ, USA), nucleolin (sc-13057, Santa Cruz, Dallas, TX, USA), p62 (GP62-C, Progen, London, United Kingdom), LC3 (NB100-2220, Novus, Centennial, CO, USA), Mdm2 (AF1244, R&D Systems, MN, USA), mono/poly-Ubiquitin (clone FK2, PW8810, Affinity Research Products Limited San Francisco, CA, USA), β-Actin (127 M4866 V, Sigma, Santa Fe, NM, USA), β-Tubulin (T4026, Sigma, Santa Fe, NeNM, USA), and GAPDH (Glyceraldehyde-3-Phosphate Dehydrogenase, sc-32233, Santa Cruz, Dallas, TX, USA). Immunoreactive bands were visualized using enhanced chemiluminescence (ECL; Amersham Biosciences, Buckinghamshire, UK) or the Odyssey Infrared Imaging System (Li-Cor Biosciences, Lincoln, NE, USA). Films were scanned with a GS-700 Imaging Densitometer and analyzed with Quantity One Software (Bio-Rad, Hercules, CA, USA), or an Odyssey Classic reader and the Odyssey software package 3.0 (Li-Cor Biosciences). Full membrane images of the gels can be found in Supplementary Materials.

### 2.4. RT-qPCR from Rat and Mouse Tissues

In the rat samples, the total RNA from cardiac and skeletal muscle was obtained from 50 mg and 100 mg of tissue, respectively, using 1 mL Tripure^®^ isolation reagent (Roche Diagnostics, Basel, Switzerland) following the manufacturer’s instructions, and quantified using a Nanodrop system (BioTek, Winooski, VT, USA). One μg of total RNA was reverse transcribed to cDNA using an M-MLV commercial kit (Invitrogen, Carlsbad, CA, USA). The reaction was set up as follows: 25 °C (10 min), 37 °C (50 min), 70 °C (15 min) and 4 °C in a Gene Amp 9700 thermal cycler (Applied Biosystems, Foster City, CA, USA), cDNA solution was diluted 1/10 and stored at −20 °C until was analyzed. Real-time PCR was performed using a LightCycler^®^ 480 System II (Roche Diagnostics, Basel, Switzerland). Each reaction contained 5 μL of LightCycler^®^ 480 SYBR Green I master mix, sense and antisense primers (0.374 μM each), and 2.5 μL of the cDNA dilution in a final volume of 10 μL. The amplification program consisted of a pre-incubation step for the denaturation of template cDNA (95 °C, 2 min), followed by 40 cycles consisting of the denaturation (95 °C, 5 min), annealing (primer-dependent temperature, 10 s), and extension steps (72 °C, 12 s). The primer sequences and annealing temperatures used were: *adrbk1_for:* 5′-CATGCACAATCGCTTTGTAGTC-3′, *adrbk1_rev:* 5′-GGTCTGAGATTCTCACATGG- 3′ (58 °C); *rpl32_for:* 5′ -CCAGTCGGA CCGATATGTGAA- 3′, *rpl32_rev:* 5′ -TCTGGCCCTTGAATCTTCTCC- 3′ (60 °C); *18 s_for:* 5′ -CGAACCTCCGACTTTCGTTCT- 3′, *18 s_rev:* 5′- GCGGTGAAATTCTTGGACCGG- 3′ (61 °C); *gapdh_for:* 5′ -ACTTTGGCATCG TGGAAGGG- 3′, *gapdh_rev:* 5′ -CCGTTCAGCTCTGGGATGAC- 3′ (60 °C).

In the mice, approximately 50 mg of frozen heart was homogenized using metal beads in a Tissue Lyser (Qiagen, Hilden, Germany) and mRNA was extracted using RNeasy Fibrous Tissue Mini Kits (Qiagen), following the instructions provided by the supplier. RT-PCRs were performed by the Genomic Facility at the Centro de Biologia Molecular Severo Ochoa (abbreviated CBMSO, Madrid), using Light Cycler equipment (Roche, Indianapolis, IN, USA). Gene expression quantifications were performed using self-designed probes purchased from Sigma and labeled with Syber Green as follows: *adrbk1_for:* 5′- CATGCACAATCGCTTTGTAGTC-3′, *adrbk1_rev:* 5′- GGTCCGAGATTCTCACATGG-3′; *hprt1 for:* 5′- TCCTCCTCAGACCGCTTTT-3′. *hprt1 rev:* 5′- CCTGGTTCATCATCGCTAATC -3′; *rps29for:* 5′- CTGAACATGTGCCGCCAGT-3′, *rps29 rev:* 5′- TCAAGGTCGCTTAGTCCAACTTAAT -3′; *18 s for:* 5′-CTCAACACGGGAAACCTCAC-3′, *18 s rev:* 5′-CGCTCCACCAACTAAGAACG-3′. qPCRs and statistical analysis of the data were performed using GenEx software. A geometric mean of three stably expressed and commonly used reference genes (*hprt, 18 s*, and *rps29*) was used for data normalization.

### 2.5. Statistical Analysis

All data are expressed as mean values ± SEM and ‘n’ represents the sample size. Statistical significance was analyzed using the unpaired Student’s t-test or one- or two-way ANOVA followed by Bonferroni’s post-hoc test. Correlation between two data samples was calculated using the Pearson correlation test. All data were analyzed using GraphPad Prism software. Differences were considered statistically significant when *p* < 0.05. The threshold cycle (Ct) values of the real-time PCR were analyzed using Genex software version 6 (MultiD Analyzes AB, Sweden), considering the efficiencies of each pair of primers, which were calculated experimentally.

## 3. Results

### 3.1. GRK2 Levels Change with Age in a Sex-Dependent Manner in the Mouse Heart and Show an Inverse Correlation with Mitochondrial Fusion Markers

**Figure 1:**
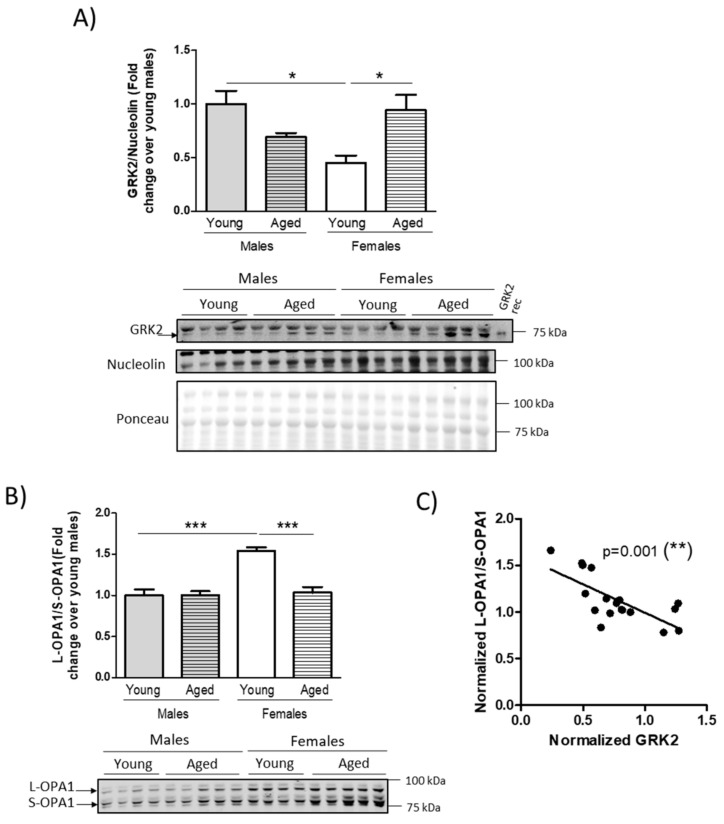
Age-associated changes in G protein-coupled receptor kinase 2 (GRK2) protein levels in the heart differ in male and female mice and inversely correlate with the mitochondrial fusion marker optic atrophy (OPA1) isoform ratio. Cardiac tissue lysates from C57BL/6 J mice of 4 months (young) or 15 months (aged) of age were analyzed by Western blot to quantify GRK2 protein levels (**A**), OPA1 isoform ratio (**B**), and nucleolin used as a loading control. Equal protein amounts among (Western blot) WB lanes was confirmed by Ponceau staining. Electrophoretic migration of molecular weight markers is indicated. Correlation between GRK2 (**A**) and the L-OPA1/S-OPA1 ratio (**B**) was assessed by Pearson’s correlation (**C**). Representative immunoblots and densitometric analysis correspond to *n* = 4–5 mice per condition. Results are represented as means ± SEM. Statistical significance was analyzed by one-way ANOVA corrected by Bonferroni’s post-test and Pearson’s correlation. * *p* < 0.05, ** *p* < 0.01, *** *p* < 0.001. GRK2 Rec, recombinant purified GRK2 protein from infected overexpressing Sf9 cells.

As mentioned above, the CVD risk dynamics show sexual dimorphism with age. Additionally, the role of GRK2 in regulating cardio-metabolic physiopathology in the heart is well established [22,23]. However, the possibility that cardiac GRK2 protein levels change with age in a sex-dependent fashion has not been addressed to date. To address this question, we analyzed the amount of GRK2 protein in the heart of young (~4 months) and aged (~15 months) male and female C57 Bl/6 J mice. We found that the GRK2 protein levels present with sexual dimorphism in the young animals and with a sex-dependent differential modulation with age. Young females had a lower quantity of GRK2 as compared to age-matched males (Figure 1A). Moreover, GRK2 levels increased with age solely in the female group, with a non-statistically significant tendency to decline in males (Figure 1A). Since GRK2 has recently been shown to modulate mitochondrial function in the heart [27,36], and mitochondrial fusion markers in High Fat Diet (HFD)-fed male mice inversely relate to cardiac GRK2 levels [31], we analyzed the isoform processing of optic atrophy (OPA1) as a readout of mitochondrial fusion capacity [37] that has been shown to be sexually dimorphic in murine models [19]. We found a higher ratio between the long and short OPA1 isoforms (L-OPA1/S-OPA1 ratio) in the young females, compared to age-matched male animals (Figure 1A). This ratio did not change with age in the male animals but we detected that it significantly decreased in females with age (Figure 1B), coherent with the previously-described changes in mitochondrial fusion detected in female mice [19]. Interestingly, we found a statistically robust inverse correlation (*p* = 0.001) between normalized GRK2 levels and the L-OPA1/S-OPA1 ratio in the cardiac tissue of our experimental group (Figure 1C). In any case, we would like to point out that although the nucleolin normalization control does not change significantly among conditions when related to the Ponceau staining as an accurate normalization procedure [38], these types of normalizations should always be considered with caution, since they are globally changing physiological conditions that may affect the levels of many proteins and gene expression patterns.

### 3.2. Modulation of GRK2 Levels in the Heart Does Not Seem to Be Mediated by Transcriptional Modulation or by Overall Changes in the Proteasomal or Autophagic Machinery

**Figure 2:**
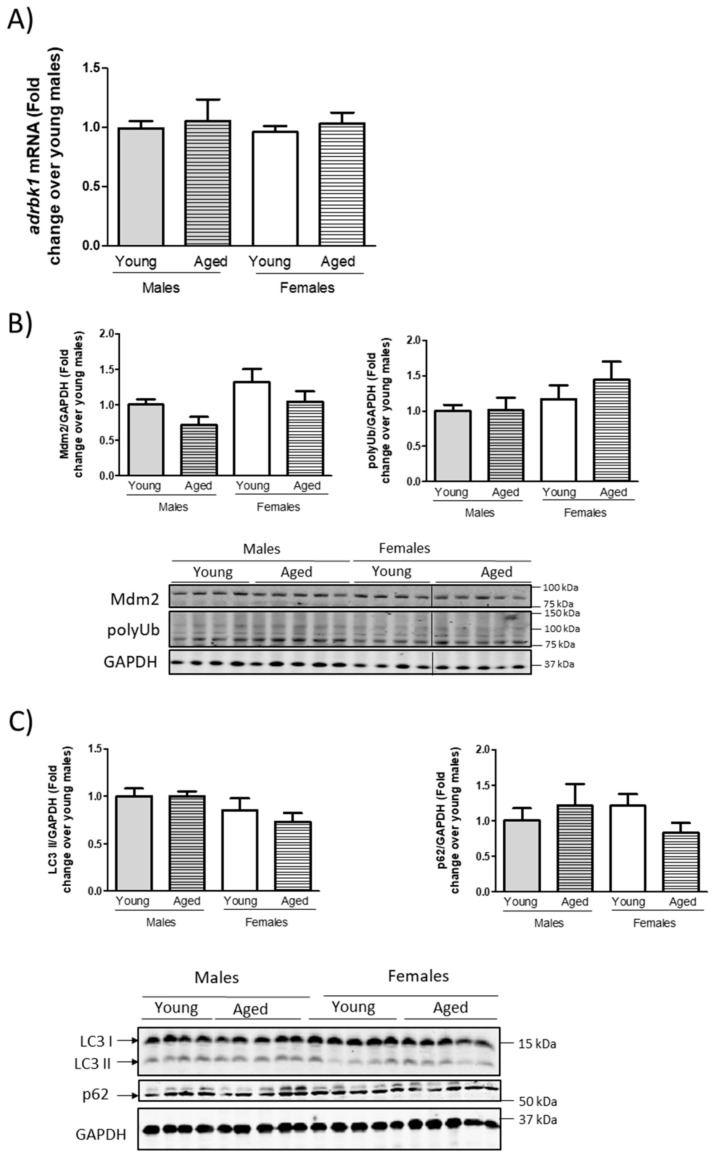
Sex-related changes in cardiac GRK2 protein levels with age are not detected at the mRNA level and overall proteasomal degradation or autophagy do not seem to be globally altered with age or sex in mouse cardiac tissue. Male and female C57BL/6 J young and aged mice were euthanized and the cardiac tissue was surgically removed and processed for RNA extraction. qPCR of GRK2 mRNA levels (*Adrbk1* normalized by a geometrical mean of *HPRT1*, *18 S*, and *RPS29*) (**A**). General markers of proteasomes (Mdm2 and mono/poly-Ubiquitin) (**B**), autophagy machinery (LC3 II and p62), and (**C**) GAPDH as a loading control were quantified by Western blot. Fold change referred to young male mice data. Results are represented as means ± SEM. Statistical significance was analyzed using one-way ANOVA followed by Bonferroni’s post-hoc test.

To further analyze how the GRK2 protein changes with sex or age, we measured the GRK2 mRNA (*adrbk1*) levels by RT-qPCR in the hearts of these animals, since the transcriptional modulation of the GRK2 dosage has been described in other contexts [22,31,39]. However, we could detect no significant sex- or age-dependent changes in *adrbk1* expression (Figure 2A), which indicates that a post-transcriptional regulation must have taken place. Since GRK2 levels have been shown to be regulated by proteasomal-dependent degradation in cardiac tissue [33] and by autophagy in the liver [34], we tested the status of general proteasomal or autophagic activity markers. As shown in Figure 2B, the levels of the ubiquitin ligase Mdm2, a key regulator of the proteasomal pathway known to be implicated in the degradation of GRK2 [40], and the levels of mono/poly-ubiquitinated proteins as a readout of global proteasomal activity, showed no statistically significant differences that could serve explain the observed changes in the GRK2. The levels of established autophagy markers such as LC3 processing or p62 accumulation (Figure 2C) displayed neither global differences in autophagy in young females vs. young males nor an autophagy blockage in aged vs. young females that could underlie age-dependent GRK2 upregulation in the hearts of aging females, although this type of analysis does not rule out that more specific variations may target defined proteins for degradation using these systems.

**Figure 3:**
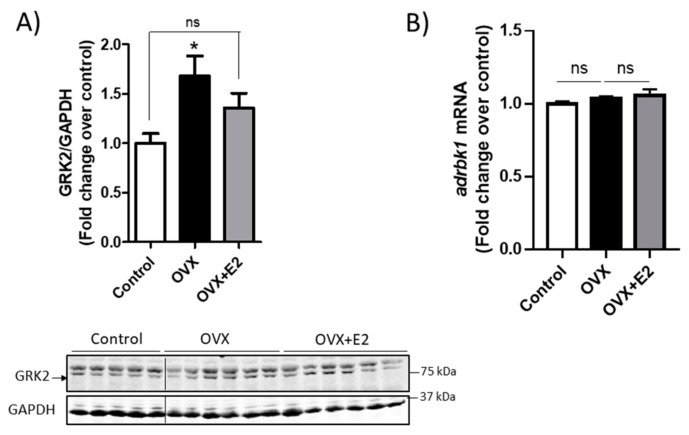
Cardiac GRK2 protein levels are modulated by sexual hormones in rats. Cardiac lysates from female ovariectomized (OVX) animals and OVX rats, supplemented with 17β-estradiol (OVX + E2), were subjected to WB with antibodies against GRK2 and GAPDH, *n* = 5–6 (**A**) or to mRNA extraction and qPCR to assess *adrbk1* levels (normalized to *rpl32* and *18 s*) *n* = 5–10 (**B**). Representative immunoblots and densitometric analysis are shown, results are represented as means ± SEM, statistical analyses were performed using one-way ANOVA followed by Bonferroni’s post-hoc test (**A**,**B**) * *p* < 0.05.

Estrogen levels modulate the GRK2 protein levels in the female heart and skeletal muscle. Sexual hormones are depleted more abruptly with age in female animals than in males, and therefore we reasoned that this differential factor might contribute to the sexual dimorphism observed in cardiac GRK2 dynamics. We thus analyzed the impact of ovariectomy and estrogen supplementation on cardiac GRK2 dosage in a murine model of female Wistar rats. We observed that the lack of ovarian function promoted an increase in cardiac GRK2 protein levels that could be partially reverted upon 17 β-estradiol (E2) supplementation (Figure 3A). Coherent with the changes reported in *adrbk1* mRNA in aging females (Figure 2A), the upregulation of GRK2 protein in the heart upon the loss of ovarian hormones did not appear to have been caused by hormone-triggered changes in the transcription of the *adrbk1* mRNA, since its levels did not change significantly upon ovariectomy (Figure 3B). Additionally, the downregulation of GRK2 protein caused by E2 was not due to transcriptional modulation since E2 does not have any effect on *adrbk1* mRNA levels (Figure 3B). Altogether, these results indicate that ovariectomy causes an increase in the cardiac GRK2 protein that can be partially reversed by estrogen supplementation and that does not seem to be caused by classical transcriptional modulation.

**Figure 4:**
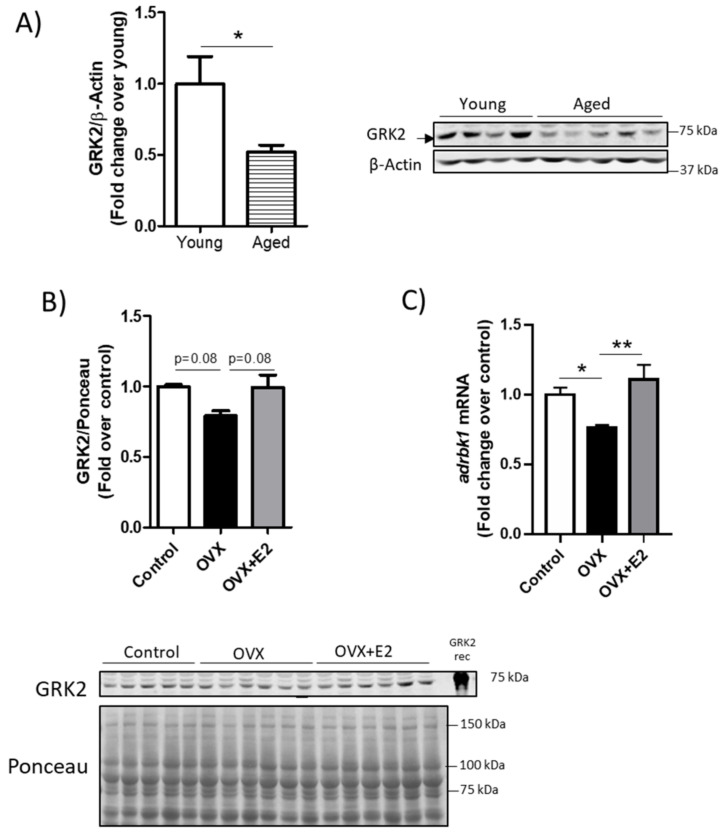
Modulation of GRK2 protein levels in females upon aging or ovariectomy in skeletal muscles is opposite to that of the cardiac muscle. Young and aged female mice were euthanized, and the soleus and gastrocnemius were surgically removed and processed for Western Blot and probed with antibodies against GRK2 and β-actin. (**A**). Effects of ovariectomy and estradiol (E2) replacement in the gastrocnemius muscle of rats probed with antibodies against GRK2 and normalized by the Ponceau staining [38], *n* = 5–6, (**B**) and in *adrbk1* mRNA levels normalized by *gapdh* as detected by qPCR, *n* = 5–6 (**C**). Representative immunoblots and densitometric analysis of 4–6 animals per group are shown. Results are represented as means ± SEM, statistical analyses were performed using one-way ANOVA followed by Bonferroni’s post-hoc test (**B**,**C**) or an unpaired *t*-test (**A**) * *p* < 0.05, ** *p* < 0.01. GRK2 Rec, recombinant purified GRK2 protein from infected overexpressing Sf9 cells.

Notably, the modulatory effect of ovarian hormones and particularly of estrogens on GRK2 levels in cardiac muscle seemed to be specific to this tissue. In the skeletal muscle of female mice, GRK2 levels decreased with age (Figure 4A), thus showing an opposite regulation to that observed in the cardiac tissue. Contrary to what occurs in the heart, in rat soleus and gastrocnemius muscles, we did not detect statistically significant changes in the GRK2 protein levels after ovariectomy or E2 supplementation, although a tendency might be observed (Figure 4B). In the skeletal muscle, ovariectomy significantly decreased *adrbk1* mRNA expression, while E2 upregulated it (Figure 4C) although these changes were not translated into changes in protein levels. These results suggest that different mechanisms might be implicated in GRK2 modulation by estrogens in the cardiac and skeletal muscles.

## 4. Discussion

GRK2 is a key regulator of cardiovascular physiopathology [22,23], and changes in the GRK2 levels in the heart have been reported in different pathological situations, including heart failure, hypertension, and lipid overload. Conversely, the expression of GRKs is thought to be unaffected by age (reviewed in [41]). However, few studies on this topic have been performed on females. In this work, we determined that aging induces an increase in GRK2 levels in the heart only in female mice, and that cardiac GRK2 protein levels are sensitive to ovarian hormones. Interestingly, this increase in GRK2 levels may participate in the loss of protection against CVD found in females after menopause.

GRK2 protein levels in the heart parallel the differential sex- and age-dependent sensitivity to the development of CVD. GRK2 levels are lower in young female mice, as compared to age-matched males, and they increase significantly with age solely in females, thus correlating with the loss of protection reported after menopause in both human and animal models of CVD [4,5]. Moreover, the lower GRK2 dosage observed in the young female heart inversely correlates with an increase in mitochondrial fusion markers such as the L-OPA1/S-OPA1 ratio, which strengthens the relationship between GRK2, the modulation of mitochondrial dynamics, and mitochondrial function [27,31,36,39]. Thus, a characterization of the mechanisms underlying the sexual dimorphism observed in age-induced regulation of cardiac GRK2 stands as a relevant question that may have consequences in preserving cardiovascular health, in particular in postmenopausal females. We show here that ovarian hormones and, in particular, estrogens have a clear impact on GRK2 protein levels.

Estrogen levels decline after menopause and constitute the main contributor to the increased CVD risk observed in females with age [12,15,42]. The observed benefits conferred by estrogen supplementation support the use these strategies as feasible therapies in both animal models [8,9,11] and early post-menopausal patients [12,13,14,15,43]. Both the classical estrogen receptors (ERα and ERβ) and the plasma membrane GPR30 or the GPER receptor (a member of the GPCR superfamily) have been reported to be implicated in the protection of the heart by regulating gene expression through their nuclear actions as well as by modulating the cardiomyocyte signaling pathways (reviewed in [12,43,44]). In our model, cardiac GRK2 levels increased in rats upon ovariectomy, whereas estrogen supplementation partially reversed this increase. These results demonstrate that ovarian hormones, and particularly estrogens, are able to modulate GRK2 dosage in the heart.

Interestingly, we found a different modulation of GRK2 by ovariectomy and estrogen supplementation in the skeletal muscle. We observed an opposite modulation of GRK2 between the skeletal and cardiac muscles in females with age, with a downregulation taking place in the former and an upregulation being observed in the latter. It is thus tempting to suggest that this differential modulation of GRK2 might have been caused by the occurrence of the different tissue-specific regulatory mechanisms triggered by ovarian hormones/estrogens which would be dependent on the muscle type. These modulations could potentially occur by way of differential activation of the classical nuclear receptors and/or the cytoplasmic actions of GPER that might mediate both the genomic and non-genomic actions and ultimately impinge upon GRK2 modulation [45,46]. Estrogens have been previously described as upregulating GRK2 levels in the brain [47] and breast cancer cells [48], results that are in line with our data concerning the skeletal, but not in the cardiac, muscles. Together, these observations may indicate that the regulation of GRK2 by ovarian hormones and estrogens is controlled in the heart by tissue-specific mechanisms that are not common to other organs or cell types. Particularly in the heart, these mechanisms appeared to implicate post-transcriptional modulation without involving global changes in autophagy or proteasome activity, at least as observed using the general markers of these processes. However, we cannot discard the possibility that estrogens might be activating or inhibiting these or other cellular processes in a more local or target-specific manner, since estrogens have been determined to finely tune the activation of the autophagic and proteasomal machinery in other tissue and cell types through their modulatory effects on different pathways [49,50].

Previous evidence supports the notion that increasing GRK2 dosage or activity could be detrimental for cardiovascular health due to its role as a negative regulator of the GPCR and non-GPCR-mediated cascades and of insulin responses (see [22] for a review). Likewise, interfering with GRK2 by decreasing its levels or inhibiting its kinase activity can be protective [23,26,29,51,52]. Interestingly, GRK2 has also been reported to modulate not only mitochondrial functionality [27,36,53], but also mitochondrial biogenesis and dynamics [31,39]. Estrogen actions, specifically through GPER, can regulate mitochondrial dynamics by promoting mitochondrial fusion [10,54,55] which correlates with better mitochondrial functionality and cardiac health in young females [10,19], and which directly contributes to sex- and age-dependent differences in cardiac health and metabolism [56,57,58]. In fact, mitochondrial dynamics are emerging as a core player in cardiovascular homeostasis, with their impairment being linked to myocardial damage and cardiac disease progression [16,59,60]. Specifically, increased mitochondrial fusion has been shown to benefit mitochondrial functionality by favoring optimal metabolite utilization in oxidative phosphorylation and reactive oxygen species handling, which directly affects cardiac metabolism and health [17,37,61,62]. Given the inverse correlation observed between GRK2 and mitochondrial fusion markers such as OPA1 processing, it is tempting to suggest that the GRK2-mediated modulation of this process could contribute to the increased mitochondrial functionality reported in young male vs. female mice [19] that is later lost in females upon estrogen decline [10,63].

**Figure 5:**
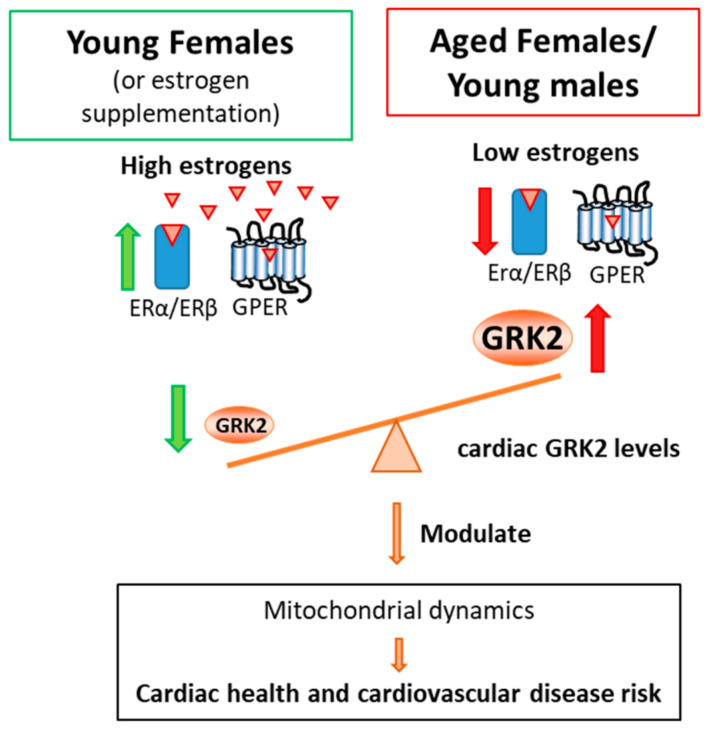
Schematic representation of GRK2 modulation by estrogens in the heart and its parallels with cardiovascular risk. In young female animals, high estrogen levels contribute to maintaining a lower GRK2 dosage whereas, in age-matched males or aged females with lower estrogen levels, higher GRK2 protein levels are detected. Elevated GRK2 levels would decrease GPCR-mediated signaling, and potential estrogen-mediated effects, negatively impinging on mitochondrial fusion markers such as OPA-1 processing which inversely correlate with GRK2 dosage. Thus, the GRK2-mediated modulation of mitochondrial dynamics might potentially contribute to sex- and age-dependent differences in mitochondrial functionality and cardiac health.

Our data suggest that the lower GRK2 dosage found in young female mice, as well as its upregulation with age, could contribute to the age-biased sensitivity to the development of CVD observed in women and female models [4,5,7,64]. We show here that the estrogen decline that occurs upon menopause could contribute to the increase in GRK2 observed in aging females, as suggested by the results in our ovariectomy model, although the possible contribution of other ovarian hormones cannot be discarded. In fact, it is tempting to speculate that decreased estrogen action and upregulated GRK2 levels could potentially reinforce each other by establishing a vicious circle. Upregulated GRK2 (with age or as a consequence of comorbidities as obesity [31]) could desensitize the GPER estrogen receptor as has already been suggested [65], through its canonical actions on GPCRs. In this potential scenario, estrogen signaling would in turn be impaired, becoming inefficient at keeping GRK2 levels at bay in the heart. As a consequence, the GRK2 levels would remain upregulated, thus fostering its known maladaptive impact on cardiac function and metabolism [22,23]. Also, the GPER-mediated estrogen protective actions would be further decreased, which could worsen cardiac health, as observed in postmenopausal females (see Figure 5). Given the global burden of CVD, specifically among women, a better understanding of the regulatory loops that could take place between GRK2 and estrogens could potentially help improve the treatment of post-menopausal patients. Thus, this study may establish a proof-of-concept that selective therapies based on GRK2 inhibitors, ideally combined with other therapeutic strategies, might be helpful in the prevention and treatment of CVD, particularly in female patients.

## Supplementary Materials

The full uncropped images of the Western Blots are available online at https://www.mdpi.com/2073-4409/10/3/673/s1.

## Nonstandard Abbreviations

CVD, cardiovascular disease; E2, estrogen (17 β-estradiol); ER, estrogen receptor; GPCR, G protein-coupled receptor; GPER, G protein-coupled estrogen receptor; GRK2, G protein-coupled receptor kinase 2; OPA1, optic atrophy 1; OVX, ovariectomized (rats).
