# Supplementary material for: Cardiac GRK2 Protein Levels Show Sexual Dimorphism during Aging and Are Regulated by Ovarian Hormones
Source: Cells. 2021 Mar 17;10(3):673. doi: 10.3390/cells10030673 (PMC8002941; doi:10.3390/cells10030673)
Supplement: Supplementary file 1 [file cells-10-00673-s001.pdf]

Figure S1

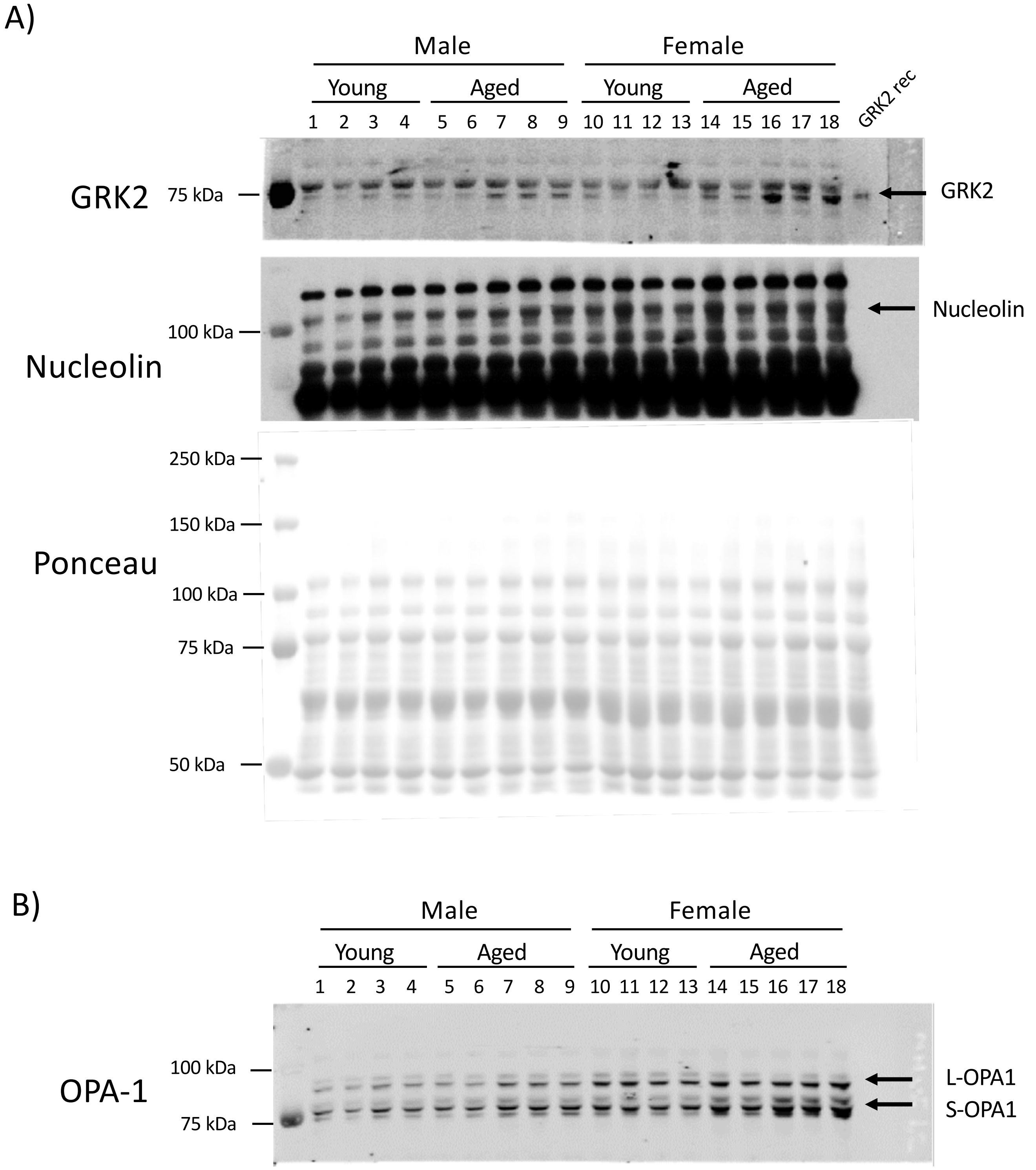

The same membrane was used for the serial incubation with GRK2, nucleolin and OPA-1 (in order of incubation).

"GRK2 rec" stands for recombinant GRK2 purified from infected overexpressing Sf9 cells

Figure S2

B)

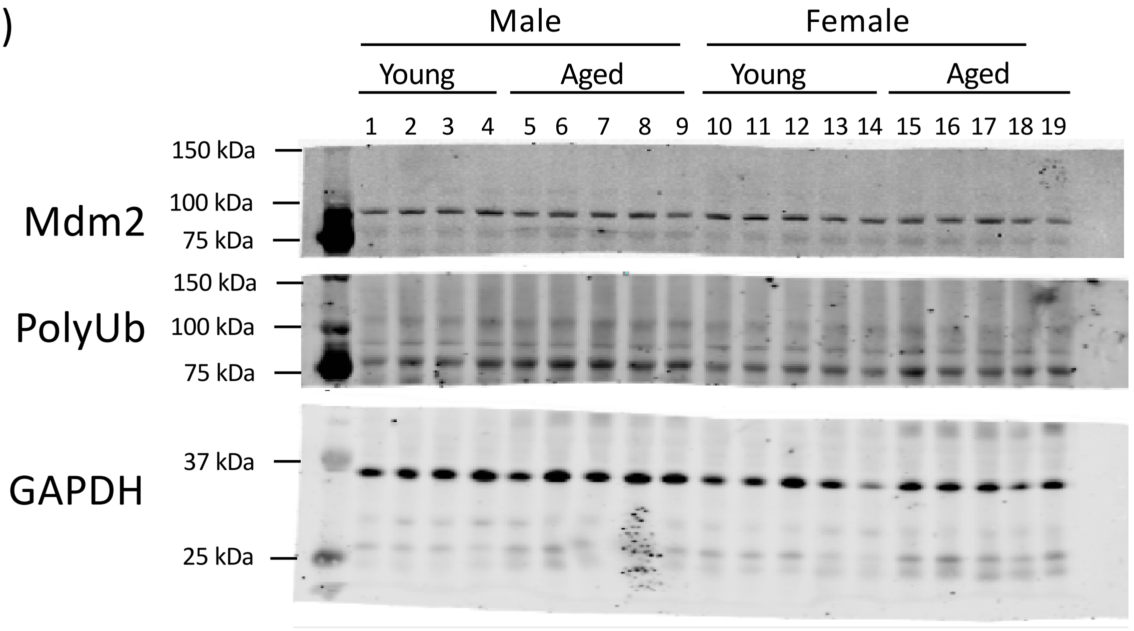

C)

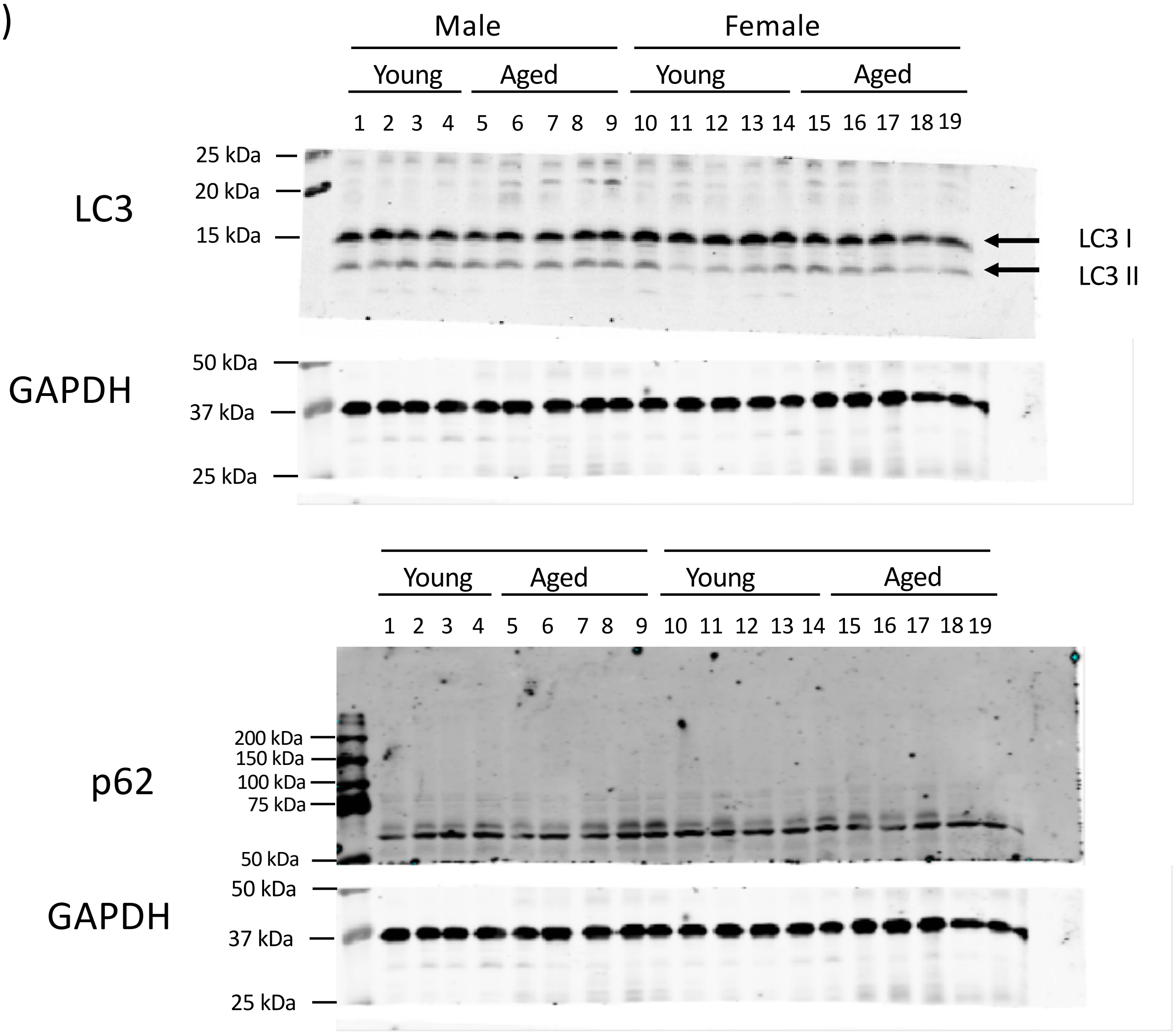

LC3, p62 and GAPDH were blotted in different membrane fragments of the same gel.

Figure S3

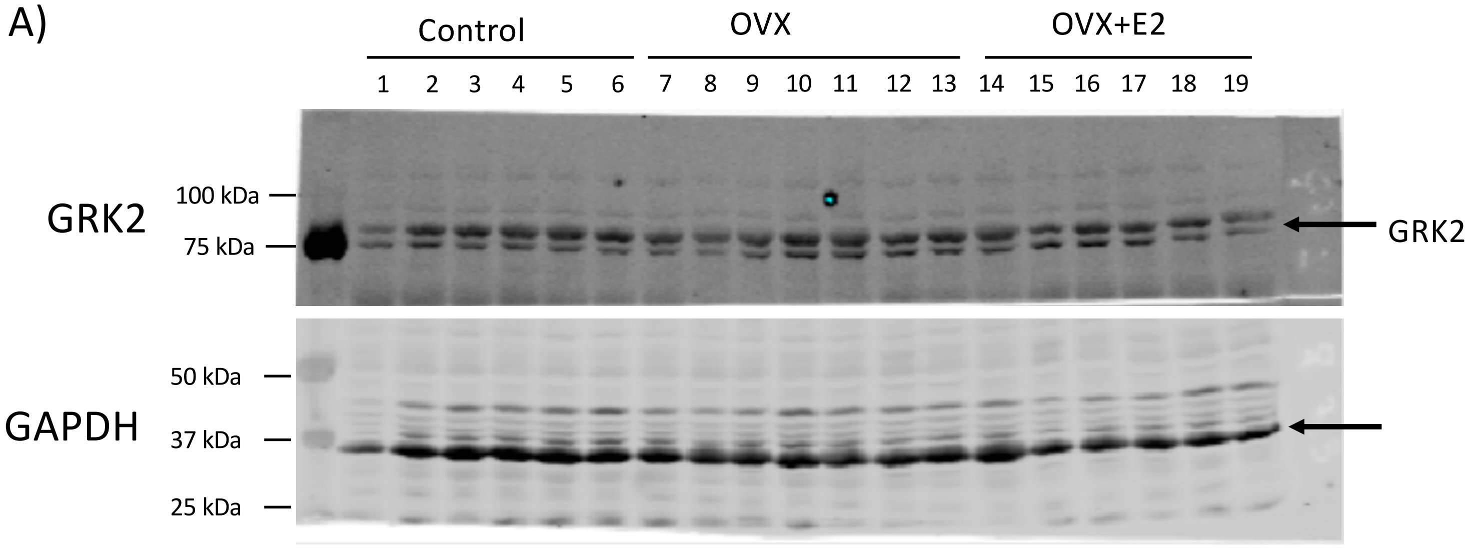
A)
Control
OVX
OVX+E2
1 2 3 4 5 6 7 8 9 10 11 12 13 14 15 16 17 18 19
GRK2
100 kDa
75 kDa
GRK2
GAPDH
50 kDa
37 kDa
25 kDa

Figure S4

A)

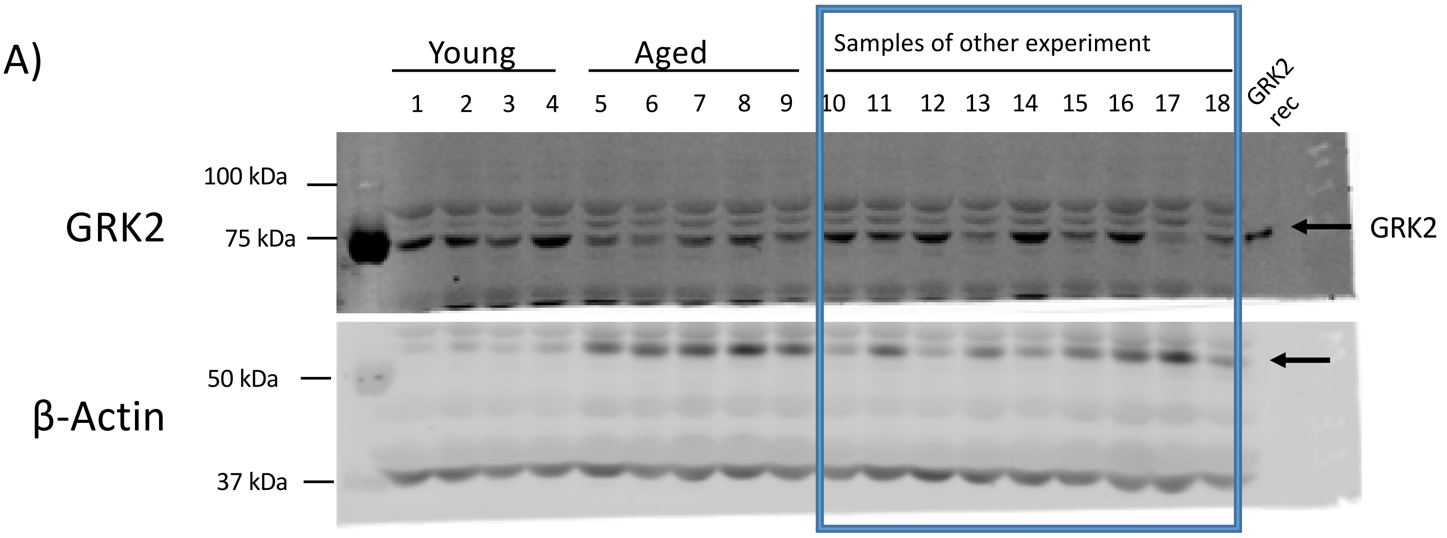

Lanes 10-18 (inside the square) belong to samples included in another experiment out of the scope of this article.

B)

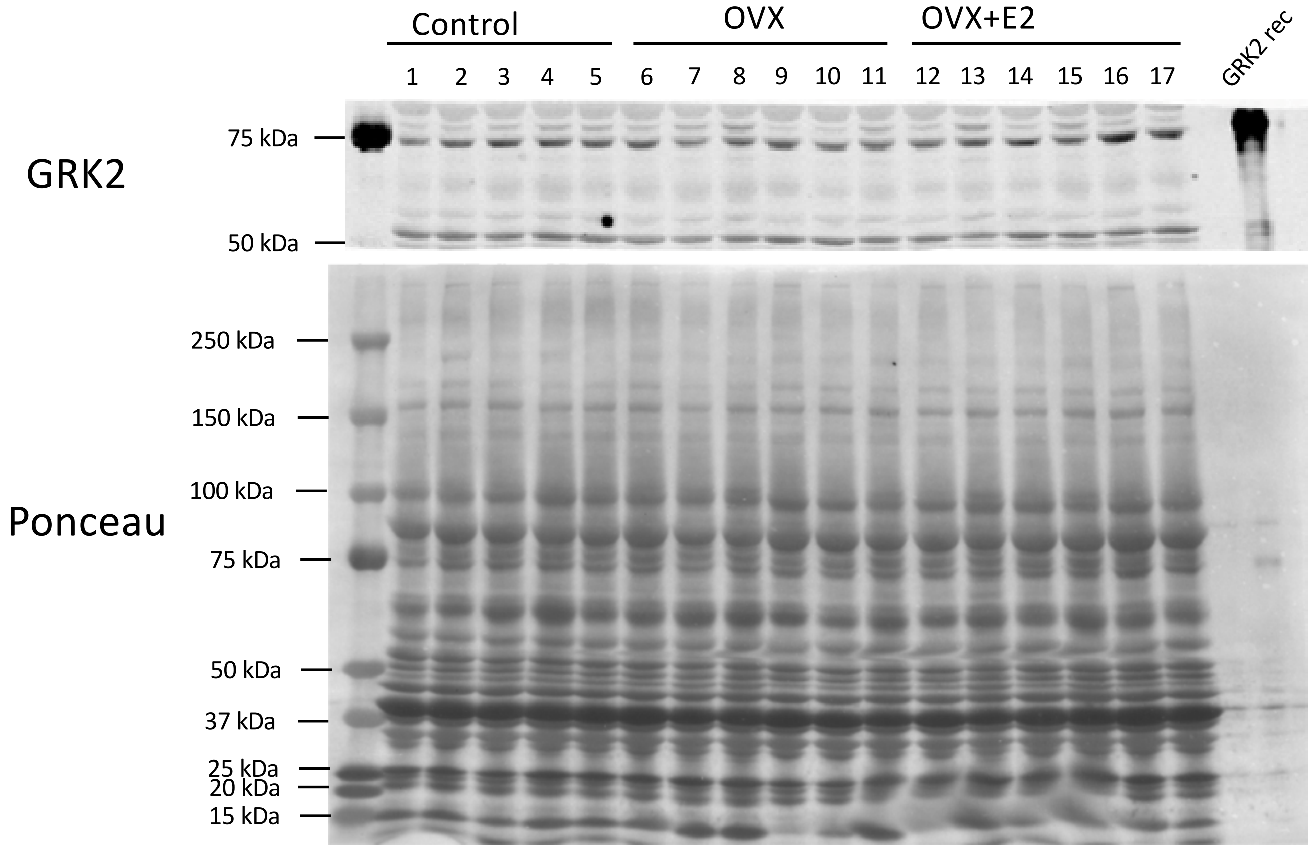

"GRK2 rec" stands for recombinant GRK2 purified from infected overexpressing Sf9 cells
